# Re-socializing pre-health education in the context of COVID: Pandemic prompts for bio-social approaches

**DOI:** 10.3389/fmed.2022.1012821

**Published:** 2022-11-02

**Authors:** Emma Mitchell-Sparke, Katharyne Wilcox Mitchell, Matthew Brookman Sparke

**Affiliations:** ^1^School of Medicine, Tufts University, Boston, MA, United States; ^2^Department of Sociology, University of California, Santa Cruz, Santa Cruz, CA, United States; ^3^Department of Politics, University of California, Santa Cruz, Santa Cruz, CA, United States

**Keywords:** neoliberalism, pre-health education, pandemic, bio-social, structural competencies, community health, global health, community partnerships

## Abstract

COVID-19 has underlined the critical importance of bringing biosocial and biopsychosocial approaches to pre-health education. Given the striking social inequalities that the pandemic has both exposed and exacerbated, we argue that bridging between the biomedical and social sciences with such approaches is now more appropriate and urgently needed than ever. We therefore call for the re-socialization of pre-health education by teaching to develop socio-structural competencies alongside physical and biological science knowledge. We suggest that community partnerships, which address local inequalities and their global interdependencies, should be encouraged as an essential element in all pre-health education. Educators should also support such partnerships as opportunities for students who come from more minoritized and impoverished social backgrounds to see their own social knowledge–including community-based knowledge of health-injustices revealed by the pandemic–as the basis of biopsychosocial expertise. By prioritizing this reconceptualization of pre-health education, we can empower future health workers to prepare more adequately for future health crises in ways that are socially aware and structurally transformative.

## Learning from the pandemic as a bio-social X-ray

Like an x-ray of broken social structures around the world, the COVID-19 pandemic has repeatedly exposed social causes of ill-health (1). It has also exacerbated these same social vulnerabilities, showing how a virus can recursively exploit and extend the structural fault-lines in societies that make poor and minoritized communities especially prone to higher rates of infection, hospitalization, and death. COVID-19 has thereby revealed the complex convergence of biosocial, psychosocial and biological factors in disease co-pathogenesis, in the process providing innumerable x-ray-like depictions of what Marya and Patel (2) call “the anatomy of injustice.” At the same time, it has further highlighted how dominant political-economic interests and social power relations condition the way that societies and health systems respond–and fail to respond–to health crises (3–5).

The convergence of neoliberal capitalism and structural racism with the virus has led to especially damaging approaches to the disease. These have included the abandonment of minoritized communities to the pandemic in rich countries, while at the same time condemning people in the Global South to vaccine apartheid (6, 7). COVID has widened disparities in already far-from-universal healthcare coverage, while simultaneously imposing additional care burdens on women and people of color working in the care sector (8–11). In the US context, where we have seen excess death accepted as the price for “liberating” the economy from public health regulations, lack of interest in the wellbeing of essential workers has led to especially disproportionate disease burdens for BIPOC communities (12, 13).

These observations vindicate early “socializing” arguments about the need for pandemic responses that “address the social contexts driving its distribution and burden” [(14), p. 1]. Some scholars have articulated the critical importance of a specifically biopsychosocial understanding of the COVID crisis, emphasizing the mental health challenges brought on by the pandemic (15). By acknowledging these challenges as biopsychosocial realities, Kop argues that health workers are better able to understand the interrelatedness of patient risk factors and the complex nature of illness (15). Other scholars, including in *Frontiers in Medicine*, have highlighted the need to attend to how disabilities have widely curtailed access to basic information about the pandemic (16).

Here, we take up these re-socializing and information-sharing challenges by focusing on educational responses catalyzed by COVID. In particular, we are concerned with the implications of the pandemic for what is widely referred to in US universities and colleges as “pre-health education.” This term refers to the mix of science, social science and health classes that students are expected to take (along with co-curricular activities) as preparation for applying to professional schools of medicine, dentistry, pharmacy, public health, and associated allied health programs.

For students who want to pursue healthcare careers, the conceptualization of health that [(14), p. 2] identify in social medicine–an approach “that unites the social, economic, and biological”−is especially germane. This is especially true in our contemporary globalized world, which has led to the complex, intersectional, and uneven nature of disease illuminated by the COVID pandemic (17). While the “biopsychosocial” is a concept that has now been firmly established in healthcare delivery, and while biosocial approaches have been advanced in global health, a push to re-socialize health *education* more widely still remains to be made despite important advances in what Marya and Patel call the “anatomy of injustice” (2). This means drawing on these closely related literatures to highlight and promote the forms of learning critical for students as they begin their health journeys through college.

If COVID can be studied as an x-ray revealing the diverse social causes of health vulnerability and health system failure, we think it is imperative to give our students the kind of education that enables them to read and understand these images in a holistic way. If more students are educated in such a manner at the undergraduate level, more health workers will be better able to respond to the biosocial co-pathogenesis of future health crises than has been witnessed in the inadequate responses to the COVID pandemic, both at national and global levels (17). In the paper, we first point to key texts and terms that can serve as important resources for advancing biosocial learning in pre-health education. Secondly, we recommend ways to support student self-reflection and meta-learning that centers the students' own experiences, including community-based participatory research, pandemic service, and personal backgrounds within underserved and minoritized communities, as the basis of biosocial expertise.

## Re-socializing pre-health pathways with socio-structural competencies

Biosocial and biopsychosocial approaches re-socialize illness as the embodiment of diverse social pathologies, or as what Paul Farmer critically referred to as “pathologies of power” (18). Farmer and his colleagues at Harvard subsequently drew on this important concept to reimagine the teaching of global health, putting disease challenges such as AIDS, TB, and Malaria into contexts of emergence deeply scarred by the political and economic pathologies of neoliberalism. “Microbes such as HIV and *Mycobacterium tuberculosis*,” they explain, “cannot be understood properly at the molecular, clinical, experiential, or population level, without analysis spanning the molecular to the social” [(19), p. 9]. A purely biomedical model would miss many of the factors critical to the spread of the diseases, including the ways that health systems were widely undermined by austerity, as well as the high costs of anti-retrovirals made unaffordable by a mixture of patents and user fees. By contrast, in attempting to provide their scale-spanning biosocial analysis, Farmer et al. examine how the social determinants of neoliberal policy–from the macro-economic austerity imposed by structural adjustment programs to the micro-economic calculus of cost-effectiveness analysis used to ration care–actually drove AIDS and TB as pandemics.

Like AIDS and TB before it, COVID has exploited societal weaknesses created by neoliberal policy regimes, including the manner in which societies have been restructured by social inequality and health systems undermined by market rule (4, 20–22). The pandemic revealed that, while we are all biologically vulnerable as *human beings*, our varied conditions of *being human* lead to vast variations in vulnerability. It has also illustrated through policy-based outcomes such as vaccine apartheid how the health of some continues to be socio-legally privileged over the health of all through neoliberal market rules. Such market rules include the World Trade Organization's (WTO) protections for intellectual property (IP) patents under the Trade-Related Aspects of Intellectual Property Rights (TRIP) rules, despite repeated demands during the pandemic for a meaningful waiver from the associated IP monopolies (23).

Another teachable biosocial concept introduced by Farmer is “structural violence” (24). This concept provides a visceral understanding of social structures inflicting violence on the bodies of those who have been dispossessed. “Structural violence,” he maintained, “is structured and stricturing. It constricts the agency of its victims. It tightens a physical noose around their necks, and this garroting determines the way in which resources—food, medicine, even affection—are allocated and experienced” [(24), p. 315]. In the COVID context, “structural violence” as a biosocial conceptualization of co-pathogenesis is particularly useful. This is a context where we have seen multiple social structures−neoliberal policy norms, structural racism, and patriarchy prominent among them−converging with a virus in ways that have killed millions of people (25). Providing students with a vocabulary for describing such co-pathogenic convergences with a sensitivity to social structure is a vital competency to add to their pre-health training.

Considered this way, the terminology of structural violence also aligns with another useful set of tools introduced to health education by Hansen and Metzl under the banner of “structural competency” (26). Focused more narrowly on the racist structures of structural violence within the United States, Hansen and Metzl have called for structural competency training in pre-health and medical education as a way of critically augmenting more dominant pedagogic programs defined in terms of teaching “cultural competency” (26). Like Farmer, they argue that physicians must be able to trace illness to its distal social determinants, hence their call for an education that fosters structural competencies about systemic racism within particular national and regional contexts (26).

They further insist that with a deeper understanding of the ways in which social, historical, political, and economic forces shape health, students can become health workers and physicians who can better address patients' needs by focusing their efforts on structural interventions (27). By working with local NGOs and policy makers to improve unequal social conditions, for example, they should be able to provide care more effectively. If deprived of an integrated and interdisciplinary biosocial education in structural competency, future health workers are ill-prepared to recognize and respond to the fundamental causes of patients' ill health, to the uneven implications of patient narratives, or to the complex politics that govern the spaces of health delivery in contexts contoured by forces ranging from austerity and biomedical gentrification to the exclusionary effects of vaccine apartheid (28–30).

## Re-socializing community experience with socio-structural competencies

While some medical schools are already making curricular shifts in response to calls for structural competencies, *pre*-health pathways for undergraduates in most US universities and colleges remain constrained by pre-COVID concepts of what is required for student success. In order to prepare a competitive application to medical school, students are generally told that they must earn high scores and grades in eight required natural science courses, as well as spend significant time volunteering, participating in scientific research, shadowing doctors, and joining extra-curricular activities related to health care (31, 32). This leaves little “extra” time for other forms of learning considered to be “outside” of the traditional biomedical pathway. When students do engage in volunteering or stints of global health service, the combination of time constraints and inadequate educational preparation can result in poor learning outcomes. Service learning and volunteering instead become boxes to be checked off or a line on a resume, rather than carefully contextualized and enduring engagements with communities that include socio-structural learning that is attentive to social power relations and community struggles and successes.

As medical humanities scholars Fletcher and Piemonte have highlighted, the resulting calculus of skills and completed tasks in pre-health education bears all the competitive hallmarks of a neoliberal social sorting system, in which students “come to view themselves in economic terms as neoliberal subjects whose efforts in service-learning can be quantified and marketed toward the end of receiving admission into competitive programs” (33). Fletcher and Piemonte argue that the best response to these problems is to develop stronger community relationships and foster enduring ties, authentic collaborations, and ongoing self-reflection as antidotes to the neoliberal norms of individualism, competition and self-branding. We see this multi-dimensional response as a powerful pedagogic corrective precisely because it is a re-socializing response. As such, it aligns with two additional and intersecting re-socializing advances in pre-health education that we have seen in the context of COVID: (1) re-locating socially-engaged participatory research locally in place of short-term global health trips; and (2) re-valorizing students' community-based backgrounds and experiences of health injustice as the basis for biopsychosocial expertise (34–37).

The COVID context has meant that, in the words of Yale students Chu et al. (38), “domestic research opportunities have been created as alternatives for students to continue gaining global health learning competencies.” Like Chu et al. we see an upside in these developments, with benefits accruing because of how “local opportunities occur in a familiar healthcare system and reduce cultural and language differentials …, allow[ing] students to remain engaged for extended periods, ensuring proper knowledge translation and communication of findings back to the community” (38). These advantages are fundamentally re-socializing in character. For the same reason, as Chu et al. also argue, they are more likely to work well when accompanied with responsible biosocial education around the social contexts and bioethics of health research. This education includes illuminating the dangers of research data extractivism and the importance of prioritizing the needs of communities over those of student self-enhancement in wealthy universities.

That said, we want to underline in addition that COVID has shown how community needs and student needs for health justice are also often aligned. This is because many pre-health students now increasingly come to higher education as first-generation students from historically minoritized and underserved communities. While the losses they have seen in their own communities in the pandemic have been extraordinarily destructive and disruptive, they have also been profoundly educational, providing yet more insight into the social pre-conditions of suffering, disease, and death. We submit that the challenge and opportunity for all health educators in this context is to enable these same students to re-valorize their community-based experiences of health injustices as a form of biopsychosocial expertise.

## Discussion and further considerations

Attuned to the ways that the COVID pandemic has become a kind of x-ray of social pathologies, we have argued that pre-health education must offer biosocial and biopsychosocial approaches that–by being more adequately interdisciplinary and re-socialized–enable students to better come to social terms with the complexity of disease co-pathogenesis. The overall objective of pre-health education should be to bring together social science approaches with natural science and technical education in ways that enable community experiences and insights about local health inequities to be recontextualized with global biosocial and biopsychosocial awareness. By relocating community-based research by students, and re-valorizing what they know from their own community experiences of health injustice, we suggest that it is possible to create re-socializing antidotes pre-health education, which remains biomedically focused.

For the same reason, we concur with other social scientists who have, in the context of COVID, endorsed “experiential knowledge alongside traditional evidence types” as a way of extending beyond the limits of scientism (39). By allowing pre-health students to also become social scientists, we argue that we can enable 21^st^ century students to come to terms with what Nikolas Rose once called “the politics of life itself” under so-called advanced liberalism (40). Attuned to bio-inequalities, such an approach should further make it possible to rethink “biological citizenship” as Rose conceptualized it, with concern for all those disenfranchised of health rights by structural violence. The lessons of COVID can in this way extend to examining the vast global spectrum of “biological sub-citizenship” that the pandemic has shown to be so stratified by the unequal social power-relations of advanced neoliberalism (4).

The COVID-19 pandemic represents just one health crisis catalyst for this kind of critical reckoning. It has been preceded by many other crises, including the devastating impacts of AIDS around the world. It will no doubt be followed by others in the future, including the strikingly unequal landscapes of health challenges related to the climate crisis. All of these threats expose vast inequities in vulnerability, morbidity and mortality, raising pressing biosocial questions that students need to be able to answer at once academically, practically, and personally.

## Data availability statement

The original contributions presented in the study are included in the article/supplementary material, further inquiries can be directed to the corresponding author.

## Author contributions

EM-S, MS, and KM contributed to conception and design of the study. EM-S wrote the first draft of the manuscript. KM and MS added sections to the manuscript draft. All authors contributed to manuscript revision, read, and approved the submitted version.

## Conflict of interest

The authors declare that the research was conducted in the absence of any commercial or financial relationships that could be construed as a potential conflict of interest.

## Publisher's note

All claims expressed in this article are solely those of the authors and do not necessarily represent those of their affiliated organizations, or those of the publisher, the editors and the reviewers. Any product that may be evaluated in this article, or claim that may be made by its manufacturer, is not guaranteed or endorsed by the publisher.

## References

[1] Guterres A,. Against Fragile Global Backdrop, Secretary-General Calls for ‘New Social Contract' in Spirit of Nelson Mandela, Marking International Day. (2020). Available online at: https://www.un.org/press/en/2020/sgsm20181.doc.htm (accessed September 30, 2022).

[2] Marya R Patel R. Inflamed: Deep Medicine and the Anatomy of Injustice. New York, NY: Farra, Strauss and Giroux (2021).

[3] Shamasunder S Holmes S Goronga T Carrasco H Katz E Frankfurter R. COVID-19 reveals weak health systems by design: why we must re-make global health in this historic moment. Glob Public Health. (2020) 15:1083–9. 10.1080/17441692.2020.1760915 32352911

[4] Sparke M Williams O. Neoliberal disease: COVID-19, co-pathogenesis and global health insecurities. Environ Plan A Econ Space. (2022) 54:15–32. 10.1177/0308518X211048905

[5] Williams O Yung KC Grépin KA. The failure of private health services: COVID-19 induced crises in low-and middle-income country (LMIC) health systems. Glob Public Health. (2021) 16:1320–33. 10.1080/17441692.2021.1874470 33471633

[6] Neely A Lopez T. Fundamentally uncaring: the differential multi-scalar impacts of COVID-19 in the US. Soc Sci Med. (2021) 272:113707. 10.1016/j.socscimed.2021.113707 33517126 PMC8724555

[7] Yamin A, Farmer, P,. Against Nihilism: Transformative Human Rights Praxis for the Future of Global Health Open Global Rights. (2022). Available online at: https://wwwopenglobalrightsorg/against-nihilism-transformative-human-rights-praxis-for-the-future-of-global-health/ (accessed February 25, 2022).

[8] Krupar S Sadural A. COVID “death pits”: US nursing homes, racial capitalism, and the urgency of antiracist eldercare. Environ Plan C Polit Space. (2022) 40:1106–29. 10.1177/23996544211057677

[9] Mezzadri A. Social reproduction and pandemic neoliberalism: planetary crises and the reorganisation of life, work and death. Organization. (2022) 29:379–400. 10.1177/13505084221074042

[10] Nazareno J Yoshioka E Adia A Restar A Operario D Ceniza Choy C. From imperialism to inpatient care: work differences of filipino and white registered nurses in the United States and implications for COVID-19 through an intersectional lens. Gend Work Organ. (2021) 28:1426–46. 10.1111/gwao.12657 34230784 PMC8251240

[11] Ridde V Hane F. Universal health coverage: the roof has been leaking for far too long. BMJ Global Health. (2021) 6:e008152. 10.1136/bmjgh-2021-008152 34896981 PMC8663104

[12] Riley A Chen YH Matthay E Glymour M Torres J. Excess deaths among latino people in California during the COVID-19 pandemic. Soc Sci Med. (2021) 15. 10.1101/2020.12.18.20248434 34307826 PMC8283318

[13] Newman E. COVID-19: a human security analysis. Glob Soc. (2021) 36:431–54. 10.1080/13600826.2021.2010034

[14] Trout L Kleinman A. Covid-19 requires a social medicine response. Front Sociol. (2020) 5:579991. 10.3389/fsoc.2020.579991 33869507 PMC8022438

[15] Kop W. Biopsychosocial processes of health and disease during the COVID-19 pandemic. Psychosom Med. (2021) 83:304–8. 10.1097/PSY.0000000000000954 33938503

[16] Dror A Morozov N Layous E Mizrachi M Daoud A Eisenbach N. United by hope, divided by access: country mapping of COVID-19 information accessibility and its consequences on pandemic eradication. Front Med. (2021) 7:618337. 10.3389/fmed.2020.618337 33585515 PMC7873525

[17] Jadoo A. COVID-19 pandemic is a worldwide typical biopsychosocial crisis. J Ideas Health. (2020) 3:152–4. 10.47108/jidhealth.Vol3.Iss2.58

[18] Farmer P. Pathologies of Power: Health, Human Rights and the New War on the Poor. Berkeley: University of California Press (2003).

[19] Farmer P Kim JY Kleinman A Basilico M. Reimagining Global Health: An Introduction. Berkeley, CA: University of California Press (2013).

[20] Navarro V. The consequences of neoliberalism in the current pandemic. Int J Health Serv. (2020) 50:271–5. 10.1177/0020731420925449 32380877 PMC7218352

[21] Saad-Filho A. Endgame: from crisis in neoliberalism to crises of neoliberalism. Hum Geogr. (2021) 14:133–7. 10.1177/1942778620962026

[22] Singer M Rylko-Bauer B. The syndemics and structural violence of the COVID pandemic: anthropological insights on a crisis. Open Anthropological Research. (2021) 1:7–32. 10.1515/opan-2020-0100

[23] Sparke M Levy O. Competing responses to global inequalities in access to COVID vaccines: Vaccine Diplomacy and Vaccine Charity Versus Vaccine Liberty. Clin Infect Dis. (2022) 75:S86–92. 10.1093/cid/ciac361 35535787 PMC9376271

[24] Farmer P. An anthropology of structural violence. Curr Anthropol. (2004) 45:305–25. 10.1086/382250

[25] Sparke M Vitale L. COVID's co-pathogenesis. Syndemic Mag. (2022) 1. Available online at: https://syndemic.ca/2022/02/04/covids-co-pathogenesis/

[26] Metzl J Hansen H. Structural competency: theorizing a new medical engagement with stigma and inequality. Soc Sci Med. (2014) 103:126–33. 10.1016/j.socscimed.2013.06.032 24507917 PMC4269606

[27] Hansen H Metzl J. New medicine for the US health care system: training physicians for structural interventions. Acad Med. (2017) 92:279. 10.1097/ACM.0000000000001542 28079725 PMC5540156

[28] Hansen H Metzl J. Structural competency in the US healthcare crisis: putting social and policy interventions into clinical practice. J Bioeth Inq. (2016) 13:179–83. 10.1007/s11673-016-9719-z 27178191 PMC4920691

[29] Reardon J Darling K Murray A Browe D Petronelli N Caramelli E. (2021). Just Biomedicine on Third Street? health and wealth inequities in San Francisco's Biotech Hub.” Counterpoints: A San Francisco Bay Area Atlas of Displacement and Resistance. The anti-eviction mapping project. PM press, Paperback/Hardcover.

[30] Sparke M Anguelov D. Contextualising coronavirus geographically. Trans Inst Br Geogr. (2020) 45:498–508. 10.1111/tran.12389

[31] Lin K Anspach R Crawford B Parnami S Fuhrel-Forbis A De Vries R. What must i do to succeed? narratives from the US premedical experience. Soc Sci Med. (2014) 119:98–105. 10.1016/j.socscimed.2014.08.017 25163642 PMC4252792

[32] Lin K Parnami S Fuhrel-Forbis A Anspach R Crawford B De Vries R. The undergraduate premedical experience in the United States: a critical review. Int J Med Educ. (2013) 4:26. 10.5116/ijme.5103.a8d3 23951400 PMC3742104

[33] Fletcher E Piemonte N. Navigating the paradoxes of neoliberalism: quiet subversion in mentored service-learning for the pre-health humanities. J Med Humanit. (2017) 38:397–407. 10.1007/s10912-017-9465-1 28664297

[34] Kayser C. Cultivating community-responsive future healthcare professionals: using service-learning in pre-health humanities education. J Med Humanit. (2017) 38:385–95. 10.1007/s10912-017-9456-2 28589307

[35] Sufian S Blackie M Michel J Garden R. Centering patients, revealing structures: the health humanities portrait approach. J Med Humanit. (2020) 41:459–79. 10.1007/s10912-020-09640-8 32654044

[36] Tsevat R Sinha A Gutierrez K DasGupta S. Bringing home the health humanities: narrative humility, structural competency, and engaged pedagogy. Acad Med. (2015) 90:1462–5. 10.1097/ACM.0000000000000743 25945967

[37] Richardson E. Epidemic Illusions: On the Coloniality of Global Health. (0000) Cambridge, MA: MIT Press (2020a).34278948

[38] Chu C Griffin G Williams J. Taking pause: a COVID-19 student reflection on global health research opportunities, training, and institutional reform. Front Sociol. (2022) 7:768821. 10.3389/fsoc.2022.768821 35127891 PMC8811217

[39] Atkinson S Bradby H Bondio MG Hallberg A Macnaugthon J Söderfeldt Y. Seeing the value of experiential knowledge through COVID-19. Hist Philos Life Sci. (2021) 43:85. 10.1007/s40656-021-00438-y 34185187 PMC8240427

[40] Rose N. The politics of life itself. Theory Cult Soc. (2001) 18:1–30. 10.1177/02632760122052020

